# High Incidence of Lysogeny in the Oxygen Minimum Zones of the Arabian Sea (Southwest Coast of India)

**DOI:** 10.3390/v10110588

**Published:** 2018-10-27

**Authors:** Ammini Parvathi, Vijayan Jasna, Sreekumar Aparna, Angia Sriram Pradeep Ram, Vijaya Krishna Aswathy, Kizhakkeppat K. Balachandran, Kallungal Ravunnikutty Muraleedharan, Dayana Mathew, Telesphore Sime-Ngando

**Affiliations:** 1 CSIR-National Institute of Oceanography, Regional Centre (CSIR), Kochi 682 018, India; jasnavijayan@gmail.com (V.J.); aparnasree4@gmail.com (S.A.); aswathyvk@sac.isro.gov.in (V.K.A.); kkbala@nio.org (K.K.B.); muraleedharan@nio.org (K.R.M.); dayanamathew0@gmail.com (D.M.); 2 Laboratoire Microorganismes, Génome et Environnement, UMR CNRS 6023, Université Clermont-Auvergne, 1 Impasse Amélie Murat, 63178 Aubière CEDEX, France; pradeep_ram.angia_sriram@uca.fr (A.S.P.R.); telesphore.sime-ngando@uca.fr (T.S.-N.)

**Keywords:** virus, prokaryotes, lytic infection, lysogeny, viral production, oxygen minimum zones of the Arabian Sea

## Abstract

Though microbial processes in the oxygen minimum zones (OMZs) of the Arabian Sea (AS) are well documented, prokaryote-virus interactions are less known. The present study was carried out to determine the potential physico-chemical factors influencing viral abundances and their life strategies (lytic and lysogenic) along the vertical gradient in the OMZ of the AS (southwest coast of India). Water samples were collected during the southwest monsoon (SWM) season in two consecutive years (2015 and 2016) from different depths, namely, the surface layer, secondary chlorophyll *a* maxima (~30–40 m), oxycline (~70–80 m), and hypoxic/suboxic layers (~200–350 m). The high viral abundances observed in oxygenated surface waters (mean ± SD = 6.1 ± 3.4 × 10^6^ viral-like particles (VLPs) mL^−1^), drastically decreased with depth in the oxycline region (1.2 ± 0.5 × 10^6^ VLPs mL^−1^) and hypoxic/suboxic waters (0.3 ± 0.3 × 10^6^ VLPs mL^−1^). Virus to prokaryote ratio fluctuated in the mixed layer (~10) and declined significantly (*p* < 0.001) to 1 in the hypoxic layer. Viral production (VP) and frequency of virus infected cells (FIC) were maximum in the surface and minimum in the oxycline layer, whereas the viral lysis was undetectable in the suboxic/hypoxic layer. The detection of a high percentage of lysogeny in suboxic (48%) and oxycline zones (9–24%), accompanied by undetectable rates of lytic viral infection support the hypothesis that lysogeny may represent the major survival strategy for viruses in unproductive or harsh nutrient/host conditions in deoxygenated waters.

## 1. Introduction

Oxygen minimum zones (OMZs) are unique oceanographic areas with reduced oxygen concentrations (<20 µM) that are caused by natural and anthropogenic factors [1]. Various physical processes (e.g., advection, poor ventilation, reduced circulation, turbulent diffusion, etc.) as well as biological processes (e.g., increased microbial respiration) are attributed to the formation of OMZs [2]. In the Indian Ocean, OMZs are found in both the Arabian Sea and Bay of Bengal [3]. OMZs of the Arabian Sea (AS OMZ), are some of the most intense in the world, with near-total oxygen depletion at 100–1000 m depth [4]. Poor ventilation, combined with increased demand for respiratory oxygen due to high biological production in the surface waters, and the limited supply of oxygen to intermediate waters are the key reasons for its occurrence [3,5]. Apart from the perennial open ocean OMZs, oxygen deficient zones develop over the continental shelf of India seasonally. During peak southwest monsoon, upwelling brings low-oxygen water over this shelf. The respiration of locally-produced organic matter in conjunction with strong stratification leads to intense denitrification, followed by sulphate reduction at very shallow depths [6].

OMZs contain microbial life that is adapted to thrive under oxygen-starved conditions and drives matter and energy transformations. Microbial-driven carbon, nitrogen, and sulphur transformations via anaerobic processes are well characterized in major OMZs of the world’s oceans [5,7,8,9,10]. Microbial community metabolism in these oxygen-starved waters directly impacts the nutrient and energy conversion processes, which leads to a loss of fixed nitrogen and the production of greenhouse gases, including nitrous oxide and methane [5]. The role of prokaryotes in biogeochemical cycles (e.g., anaerobic pathways of the nitrogen and sulphur cycles, anammox, etc.), as well as their diversity has been previously documented in AS OMZs. High abundance of ammonia-oxidizing archaea and anaerobic ammonia-oxidizing (anammox) bacteria, which are key players in the marine nitrogen cycle, occupy separate niches in the Arabian sea OMZs separated by large vertical segregation (>400 m) [11]. Thaumarchaeota involved in aerobic ammonium oxidation occupied the upper zone of the AS OMZ, whereas anammox bacteria occupied the core OMZ [12].

Viruses are the most abundant life forms on the Earth, infecting almost all life forms. In marine systems, the majority of these viruses infect microbes, such as bacteria, archaea, or microeukaryotes [13]. Viral lysis diverts the flow of carbon and nutrients away from larger organisms and pushes the food web towards a regenerative pathway, often referred to as the ‘viral shunt’. Viral abundance is dependent on various biotic (host abundance and composition) and abiotic variables (such as nutrient concentrations, salinity, and temperature) [14,15,16]. Viruses and their potential impacts on OMZs, such as eastern tropical South Pacific (ETSP), Saanich inlet, and the Baltic Sea are well documented [14,17,18,19,20]. The viral metagenomes from the ETSP showed little similarity viral genomes from other marine environments and there was minimal genetic overlap between surface, oxycline and anoxic core [14]. The virus to prokaryote ratio in ETSP showed fluctuations in the oxycline with a decline in the anoxic core. However, in the Baltic Sea, the frequency of virally infected cells was highest in the suboxic zones with limited lysogenic viral infection [17,18]. A recent study from the Baltic Sea redoxcline suggests that the prokaryotic viruses invest most of their resources into stress defense (i.e., strong capsids), rather than proliferation (i.e., high burst size), resulting in low virus production and low viral decay [19]. Apart from viruses, additional prokaryotic loss factor, such as protistan grazing, which can destroy up to 100% of prokaryotic standing stock, has been found to be negligible in anoxic waters [17]. A recent study has demonstrated that viral-mediated predation, gene transfer, and metabolic programming modulate the structure, function, and evolutionary trajectory of SUP05 bacteria in OMZs [20]. Viriomes from oxycline and anoxic waters have indicated the presence of viral infection and contained genes involved in biogeochemical cycles [14,20]. On the contrary to low rates of viral production in the Baltic Sea, high rates of viral lysis have been reported from marine Lake Rogoznica [21]. Weinbauer et al. (2003) used transmission electron microscopy, whereas Rastelli et al. (2016) used radiolabelled substrate incorporation method for estimating the viral production. This potentially implies that viruses have different impact on different OMZ environments and different methods from different locations could present different pictures of the potential impacts of viruses in the OMZs.

There are two reproductive pathways for viruses, namely, the active lytic infection and dormant lysogeny. The occurrence and ecology of lysogeny has been reviewed from marine environments with information putative marine prophages and emergence of gene transfer agents [22]. The significance of lysogeny in the marine environment was demonstrated with isolates and lysogenic phage production models [23]. Earlier studies on lysogeny in cyano- and bacteriophages suggest that lysogenic reproductive cycle is favoured during low host abundance and activity, and it varies according to the system productivity [24,25]. Recently, Brum et al. [26] demonstrated that temperate viruses switch from lysogeny to lytic replication with increase in prokaryotic production in response to resource availability in polar regions. Alternatively, a Piggyback-the-Winner model has been proposed of “more microbes, fewer viruses”, whereby high prokaryotic densities were related to higher proportions of temperate phages. [27]. However, it is reported that virus to prokaryote ratio fluctuations used to establish the Piggyback-the-Winner model do not directly assess virus-host relationships and could be linked to other factors that are related to viral–host dynamics, not only lysogeny [28,29,30]. More studies into the relative importance of lytic infection versus lysogeny are needed and they require substantial refinement for interpretation at regional and global scales.

Overall, there is a lack of information on lytic viral infection and lysogeny from the OMZs of the Arabian Sea, and, to the best of our knowledge, no studies have investigated the two viral life cycles simultaneously at the community level, so far. A recent study in the OMZs of the northwestern Arabian Sea has reported a high viral abundance and a high virus to prokaryotic ratio [31]. However, no information exists on viral processes necessary to assess the specific role of viruses in the productive southeastern region of the Arabian Sea. Hence, we hypothesize that viral lytic production might be an important factor contributing to prokaryotic mortality in the Arabian Sea OMZ. In order to understand the ecological role of virus-prokaryote interactions, we quantified rates of lytic infection and percent lysogeny in prokaryotic communities along the vertical gradient of the water column during the productive southwest monsoon in two consecutive years.

## 2. Material and Methods

### 2.1. Study Sites

**Figure 1:**
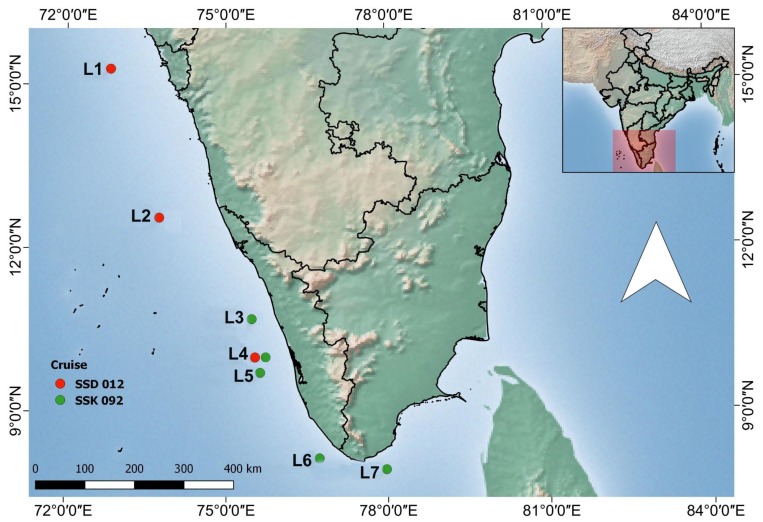
Sampling locations in the Arabian Sea. Three stations sampled during 2015 are indicated in red and six stations sampled during 2016 is indicated in green.

Water samples were collected during two oceanographic cruises onboard *RV Sindhu Sadhana* in 2015 and *RV Sindhu Sankalp* in 2016 during the southwest monsoon season (Figure 1). The cruises targeted the OMZs in the southeastern Arabian Sea, which extends from 100 m to 1000 m depth. The study area extended from 7.9° N–15.3° N to 72.8° E–77.97° E (Table 1). Samples were collected from three locations in 2015 and from five locations in 2016 (Table 1). Locations L1, L2, and L4 were sampled during 2015 and L3, L4, L5, L6, and L7 during 2016 (Figure 1). The depth of the stations ranged from 200 m to 400 m (L2: 400 m, L1, L3–L7: 200 m).

### 2.2. Sampling and Physicochemical Characteristics

Samples were collected while using Niskin bottles (10 L capacity, Hydrobios, Kiel, Germany) that were mounted on a conductivity, temperature, and depth (CTD, SBE Seabird 19, Sea-Bird Scientific, Bellevue, WA, USA) profiler rosette equipped with sensors for salinity, temperature, dissolved oxygen, turbidity, and photosynthetically active radiation (PAR). Temperature and salinity were measured while using CTD profiler (accuracy ±0.001 °C for temperature and ±0.001 S/m for conductivity). From the dissolved oxygen profiles from CTD, four sampling depths were selected representing the surface oxic layer, secondary chlorophyll maxima (SCM), oxycline and suboxic/hypoxic layers. The water samples were collected from the above depths using Niskin bottles attached to a CTD rosette. The bottles were closed during the up-cast of CTD by remote controls, starting from deepest depth onwards to minimize oxygen contamination and time delay in sub-sampling. Immediately after the retrieval of CTD on-board, sub samples for gas analysis were collected from the deepest sample onwards. Samples were withdrawn from the water sampler into DO bottles (glass 60 mL) without trapping air bubbles, allowing the bottles to overflow with at least one litre to avoid oxygen contamination. The dissolved oxygen in the bottle was fixed with 0.5 mL each of Winkler reagents and titrated on-board against standard thiosulphate using starch as the visual end point detector [32]. Dissolved inorganic nutrients such as ammonia (NH_4_), nitrite (NO_2_), nitrate (NO_3_), phosphate (PO_4_), and silicate (SiO_4_) were analysed spectrophotometrically by following standard procedures [32].

### 2.3. Abundances of Viruses and Prokaryotes Using Epifluorescence Microscopy

Biological parameters were analysed from water samples that were collected from different depths, viz, the surface, and secondary chlorophyll maxima (SCM), oxycline, and hypoxic waters based on the CTD profiles. For enumeration of viruses (VA) and prokaryotes (including both bacteria and archaea) (PA), water samples that were collected from different depths were fixed immediately with 0.02 μm filtered, buffered formalin (final concentration, 2% *v*/*v* from a 37% *w*/*v* solution of commercial formaldehyde). The subsamples (1–2 mL) were filtered (<15 KPa vacuum) through 0.02 µm pore-size Anodisc filters (Whatman, Buckinghamshire, UK) and stained with SYBR green I (Invitrogen, Thermo Fisher Scientific, Carlsbad, CA, USA; final concentration of 1:400), as previously described [33]. The filter was air dried on absorbent paper and mounted between a slide and a glass coverslip with a special antifading mountant (50% glycerol, 50% PBS—phosphate buffered saline (0.05 M Na_2_HPO_4_, 0.85% NaCl, pH 7.5), 0.1% p-phenylene diamine). When not analyzed immediately, slides were stored at −20 °C until counting under an epifluorescence microscope (Olympus BX 41). Prokaryotes were distinguished from virus-like particles (VLPs) on the basis of their relative size and brightness [33]. A blank was routinely examined as a control to check for contamination of the equipment and reagents. For both PA and VA, triplicate samples were counted and the average values were plotted (mean ± SD).

### 2.4. Viral Lytic Infection

Prokaryotic cells contained in formalin-fixed water samples (final conc. 2% *v*/*v*) were collected on triplicate electron microscope grids (400-mesh, carbon coated Formvar film) by ultracentrifugation (Optima LE-80K, Beckman Coulter SW40 Ti Swing-Out-Rotor, Brea, CA, USA) at 70,000× *g* for 20 min at 4 °C), according to Pradeep Ram and Sime-Ngando [34]. Each grid was stained at room temperature (ca. 20 °C) for 30 s with uranyl acetate (2%, pH = 4), rinsed twice with 0.02 µm-filtered distilled water to remove excess stain, and dried on filter paper. The samples were examined while using a JEOL 1200Ex transmission electron microscope (TEM) operated at 80 kV at a magnification of 20,000–60,000× to distinguish between prokaryote cells with and without intracellular viruses. A prokaryote was considered infected when at least five viruses, identified by shape and size, were clearly visible inside the host cell. At least 400–600 cells were inspected per grid to determine the frequency of visibly infected cells (FVIC). FVIC counts were converted to frequency of infected cells (FIC) while using the equation; FIC = 9.524 × VIC − 3.256 [18] and thereafter to viral-induced prokaryote mortality (VIBM), using the equation; VIBM = (FIC + 0.6 × FIC^2^)/(1 − 1.2 × FIC) [35]. Triplicate samples were counted and mean values and standard deviation values were plotted (mean ± SD).

### 2.5. Viral Production (VP)

The water samples were collected in 1 L glass bottles with narrow necks from the Niskin sampler with 1 min overflow and they were quickly closed using glass stoppers avoiding any head space or air bubbles. The filtration and experimental set up was performed in an anaerobic chamber, purged with nitrogen gas. In order to estimate VP, we used the “viral reduction method” by Wilhelm and colleagues [36]. Prokaryotes were concentrated from the water samples while using tangential flow filtration apparatus (0.2 μm pore-size polysulfone, TFF). A 100 mL of concentrated water sample was diluted with virus-free water (viruses were removed using a tangential flow filtration apparatus, CDUF001LT, Millipore, Burlington, MA, USA, with a molecular weight cut-off of 100 kDa yielding virus-free water) to a final volume 300 mL. Samples were filled into 50-mL plastic syringes and sealed with Teflon-coated butyl rubber stoppers [37] to allow for sample withdrawal without creating headspace in order to avoid the influence of the oxygen contamination in the sample. Subsamples were taken to enumerate prokaryotes and viruses at 7 h intervals over a time span of 72 h. The samples were fixed with 1% formalin for the enumeration of viruses under epifluorescence microscopy and were enumerated in the laboratory as described [33]. VP rates were determined from the first-order regression of increase in viral abundance versus time for the samples showing a single peak in viral abundance after correcting for the loss of prokaryotic hosts between experimental samples and natural samples [36] VP was calculated as VP = m × (P/b) [38], where “m” is the slope of the regression line, “b” is the concentration of prokaryotes after dilution, and “P” is the concentration of prokaryotes prior to dilution. For samples with two peaks in viral abundance, the VP was calculated while using the formula:

VP = [(Vmax_1_ − Vmin_1_) + (Vmax_2_ − Vmin_2_)]/(tmax_2_ − tmin_1_)

where “V” is viral abundance and “t” is time, and subscripts 1 and 2 refer to peaks 1 and 2 in viral abundance, respectively [39]. Triplicate samples were counted and mean values and standard deviation values were plotted (mean ± SD).

### 2.6. Induction Assays for Lysogenic Prokaryotes

Water samples in triplicates (25 mL) were treated (at time zero, t_0_) with mutagen mitomycin C (1 μg mL^−1^ final conc. Sigma, St. Louis, MO, USA) or left untreated as mitomycin controls [40]. These samples were incubated statically at in situ conditions in the dark for 24 h with subsamples being taken at every 3 h and fixed with 0.02 μm filtered formaldehyde (1% final concentration). Abundances of viruses and prokaryotes were determined by epifluorescence microscopy while using SYBR green I stain [33]. The significance of each induction vent was determined by a comparison of abundance of viruses in the mitomycin C treatment and control by an independent sample *t*-test. A statistically significant increase in the abundance of viruses in the mitomycin C treatment relative to the control indicated the presence of lysogenic prokaryotes. The frequency of lysogenically infected cells (FLC) within prokaryotic communities was calculated as: FLC = [(VA_MC_ − VA_C_)/BS × PAt_0_)] × 100, where, VA_MC_ and VA_C_ are viral abundances in mitomycin C treated and control assays respectively, after incubations. PAt_0_ is prokaryotic abundance, and BS, the maximum burst size estimated (calculated from cells that were completely filled with viruses) at the start of the experiment. Triplicate samples were counted and the mean values and standard deviation values were plotted (mean ± SD).

### 2.7. Statistical Analysis

Potential relationships among biological and environmental variables were tested by linear pairwise correlations (e.g., Pearsons correlation analysis). Principal Component or Coordinate Analysis (PCA) was carried out while using PAST software version 3.0 [41] to understand the relationship between the biotic and abiotic variables. The data was normalised by x-mean/standard deviation. Significant differences in various biotic and abiotic variables in oxic, oxycline and hypoxic waters were tested by one-way analysis of variance (ANOVA) while using PAST software. Non-parametric distance-based linear regression (DistLM) analysis was performed while using Primer 7 software, to determine the relative importance of predictor variables [42].

## 3. Results and Discussion

### 3.1. Physicochemical Characteristics of the Study Area

The Arabian Sea has unique hydrographic conditions that are modulated seasonally by physical forces, such as upwelling, winter cooling [43], and semi-annual reversal of monsoonal winds [44]. In the present study, upwelling signals were evident at L4 and L5 during 2016 (Figure S1 for L5), while they were completely absent in 2015. Brunt-Vaisala frequency plot showed a highly stable water column (>10 cycles/h) in the thermocline region with an upslope towards the surface indicating strong upwelling at L5 (Figure S1b). The temperature also showed the upsloping of isotherms towards the coastal region under the influence of strong Ekman pumping. Intense upwelling which occurs along the western coast of India during the southwest monsoon season [45,46] advances progressively from south to north [47,48]. It brings in the nutrient-rich subsurface waters to the surface to enhance the surface productivity and subsequently leads to increased particulate flux at greater depths. Such intense mineralization of this organic matter together with limited supply of oxygen to intermediate waters leads to the development of prominent OMZs at intermediate depths (100–1000 m).

**Figure 2:**
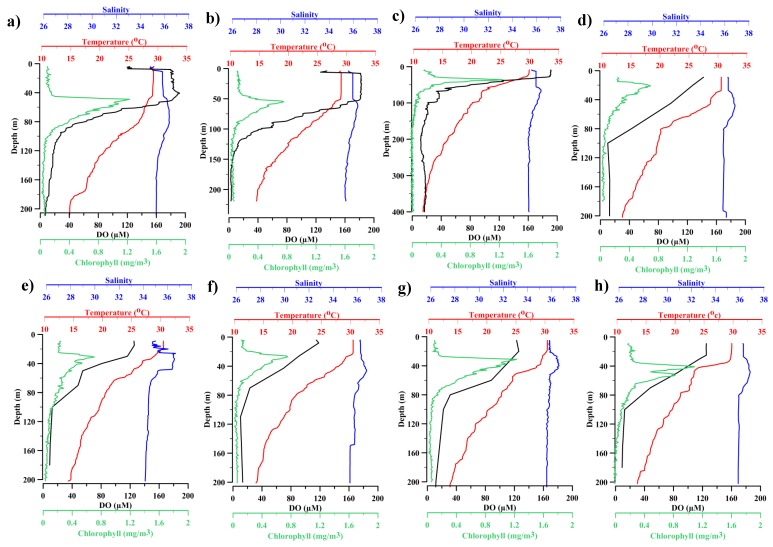
Vertical water column profiles of temperature, salinity, dissolved oxygen and Chlorophyll *a* concentrations at (**a**) L1 (**b**) L2 (**c**) L4 in 2015 (**d**) L3 (**e**) L4 in 2016 (**f**) L5, (**g**) L6, and (**h**) L7.

Vertical profiles of temperature and salinity during both of the cruises showed a highly stratified water column (Figure 2). The mixed layer depth (40–50 m) in 2015 was thicker than in 2016 (30–40 m). Subsurface chlorophyll *a* maxima (SCM, 0.56 ± 0.38 mg m^−3^) was noticed at a depth of 30–40 m at all of the stations. Temperature, salinity, and dissolved oxygen were significantly higher (*p* < 0.001) in surface than deeper waters. The concentration of dissolved oxygen (DO) was higher in 2016 (150–190 µM) as compared to 2015 (90–125 µM). However, the distribution of the physicochemical parameters was similar for both of the studied years.

Below the mixed layer, the water column was highly stratified with low concentration of DO, indicating the commencement of the oxycline layer. The oxygen concentration decreased drastically from 190 µM in the upper oxycline (40–67 m) to ~40 µM in the lower boundary of the oxycline. The oxygen concentration decreased further to 2–18 µM at the deepest depth (350 m) that was sampled. The minimum DO concentration was 2 µM at L3. Based on the DO concentration, the water column could be classified into three distinct layers, the oxic layer (141.95 ± 28.97 µM), oxycline (69.78 ± 12.47 µM), and the hypoxic to suboxic bottom layers (12.47 ± 3.38 µM). ANOVA showed that the variations in DO among these depths were significant (F28 = 3.34; *p* ≤ 0.00001). DO concentrations that were obtained along the vertical gradient are shown in Table 2.

The nitrate, silicate, and phosphate concentrations were significantly lower (*p* < 0.05) in the surface waters than in the less oxygenated waters. High nitrite concentrations (0.24 µM) were observed at SCM than surface waters (Table 2). The nitrite and nitrate values showed opposite trends with the latter showing high concentration in hypoxic waters. Nitrite values were lower in the surface waters than in the oxycline and SCM (Table 2). The silicate (SiO_4_) concentration was high in hypoxic waters (17.76 ± 8.57 µM) and low in surface waters (3.28 ± 4.52 µM). Like silicate, the phosphate (PO_4_) concentration was high in hypoxic waters (1.69 ± 0.66 µM) and low in surface waters (0.40 ± 0.47 µM) (Table 2).

### 3.2. Viral and Prokaryotic Abundance

**Figure 3:**
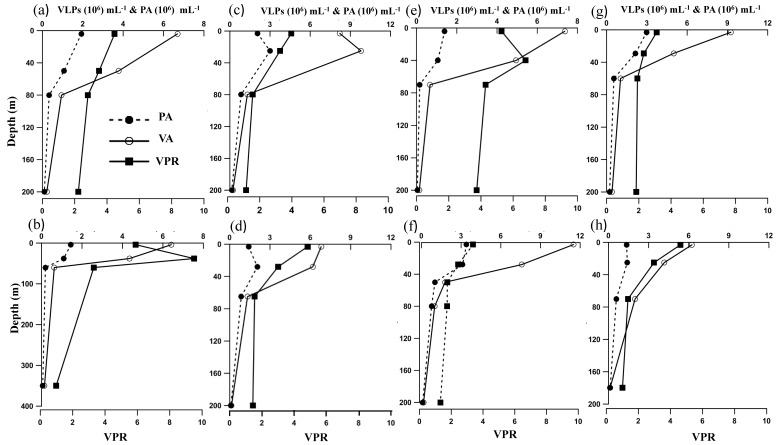
Vertical distribution of viral abundance (VA), prokaryotic abundance (PA) and virus to prokaryote ratio (VPR) at stations (**a**) L1; (**b**) L2; (**c**) L4 in 2015; (**d**) L3; (**e**) L4 in 2016; (**f**) L5; (**g**) L6; and (**h**) L7.

Viral and prokaryotic abundances were maximum in the oxic waters and minimum in the oxycline and hypoxic waters (Figure 3). Viral abundance (VA) ranged from 0.62 to 11.49 × 10^6^ VLPs mL^−1^ in the oxic waters, and 0.12 to 1.03 × 10^6^ VLPs mL^−1^ in hypoxic waters. Like VA, prokaryotic abundance (PA) was maximum in the oxic waters (0.14–3.41 × 10^5^ cells mL^−1^), decreased slightly in the oxycline (0.15–1.01 × 10^5^ cells mL^−1^), and decreased further in the bottom hypoxic/suboxic waters (0.03–0.76 × 10^5^ cells mL^−1^). ANOVA showed that these variations in VA and PA across the oxygen gradients were significant (VA, F28 = 3.34; *p* ≤ 0.00001; PA, F28 = 3.34; *p* = 0.0003). A similar trend in the distribution of viral and prokaryotic abundance was seen in both 2015 and 2016.

Virus to prokaryote ratio (VPR) was used as an indicator of predator-prey interaction between viruses and prokaryotic hosts. The VPR ranged from 1 to 10 (Figure 3). The highest VPR was recorded at the surface oxic waters (4.39 ± 0.80), which declined towards the oxycline (2.26 ± 0.96) and further beyond (1.84 ± 0.84) in the hypoxic/suboxic waters (Figure 3). VPR (~10) was significantly higher (*p* < 0.001) in the productive surface layer compared to other depths. The recent Piggyback-the winner model suggests that the viral-host relationships could be influenced by a multitude of factors that influence viral-host dynamics [27]. Hence, VPR are not invariant and their variations could be ecologically meaningful. Low VPR in the oxycline and hypoxic waters was possibly due to the low viral activity or fast viral decay, which could strongly limit the ability of viruses to find new hosts for their replication. The varying DO concentrations also alter the microbial composition, diversity, and function [49], thereby affecting the viral abundance and activity.

The distribution of viral and prokaryotic abundances showed minor variations when compared to published reports from other parts of the world. VA is present study was lower than that reported from the Arabian Sea Time Series (ASTS) station in the northwestern Arabian sea [30]. High VA in the SCM in the present study is in accordance with those observed at the bottom waters of the North Pacific [50], Adriatic Sea [51], and in Norwegian lakes [52]. However, in the oxycline, both prokayotic and viral abundances in the OMZs of the Arabian Sea were found to be lower when compared to the Atlantic, Pacific, and Mediterranean waters [52,53,54].

VA were related to physicochemical factors, such as temperature, salinity and nitrite. VA showed significant negative correlation (*p* < 0.001) with nitrite. Nitrogen and sulphur metabolism are important microbial process in OMZs. Anaerobic oxidation of ammonium with nitrite has also been identified as a dominant pathway of dinitrogen gas formation in oxygen-deficient waters [49]. However, denitrification has been demonstrated to be crucial in the removal of fixed nitrogen from the anoxic waters of the Arabian Sea [55]. A similar correlation has been reported from OMZs of the Atlantic and the Pacific Ocean [16]. Their study indicated that nitrogen and phosphorus concentrations, together with prokaryote abundances had significant effects on viral abundances, but in hypoxic environments VA was explained by a combination of physical and chemical factors.

**Figure 4:**
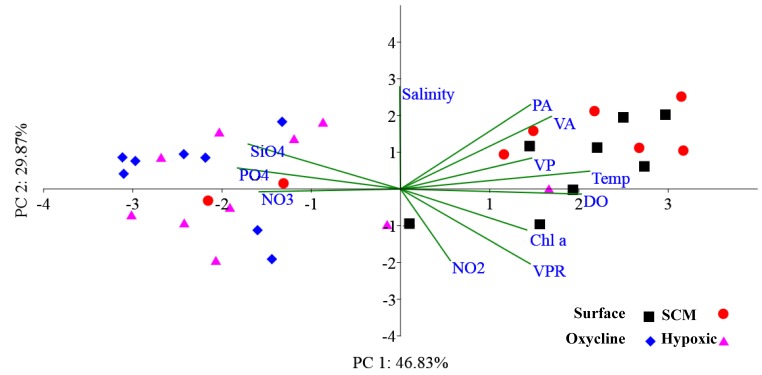
Principal coordinate analysis showing the inter-relationship between with various biotic and abiotic variables. The sampling depths are represented as different colour patterns. Abbreviations used: viral abundance (VA), prokaryotic abundance (PA), virus to prokaryote ratio (VPR), viral production (VP), Chlorophyll *a* (Chl), dissolved oxygen (DO), temperature (Temp), nitrate (NO_3_), Nitrite (NO_2_), Phosphate (PO_4_), and silicate (SiO_4_). The percentage variance explained by PC1 is 46.83% and PC2 is 29.87%.

A two-tailed correlation analysis indicated that there was significant correlation between PA and VA (Figure S2). PCA was used to demonstrate the relationship between biotic and abiotic variables (Figure 4). Other factors, such as DO, temperature, and salinity might have indirectly influenced the viral pool by altering host dynamics either by decreasing susceptibility to infection or by modifying characteristics of viral proliferation. The effects of physico-chemical and biological parameters for explaining VA was demonstrated while using non-parametric distance-based linear regression model analysis (DistLM) (Table 3). The DistLM results indicated that the most important of predictor variables for VA were DO, temperature, PA, and Chl *a* (*p* = 0.001).

### 3.3. Lytic Viral Production, Viral-Mediated Prokaryote Mortality, and Lysogenic Induction

**Figure 5:**
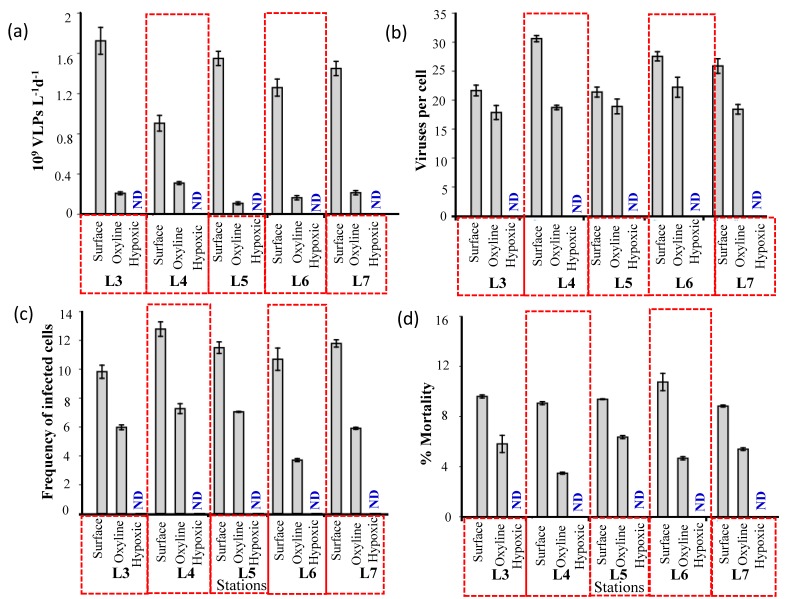
Represents the (**a**) Viral lytic production (10^9^ VLPs L^−1^·d^−1^) (**b**) Burst size (numbers per cell), (**c**) Frequency of infected cells (% IC), and (**d**) viral induced prokaryotic mortality (%) at various oxygen concentrations, surface, SCM, oxycline, and hypoxic layers at stations L3, L4, L5, L6, and L7. ND: Not detected.

To determine lytic viral production (VP) in oxygen deficient waters, the samples were incubated further in plastic syringes sealed with Teflon-coated butyl rubber stoppers to avoid the influence of oxygen contamination in the samples. In a recent cruise (SSD 049, May 2018), a similar experiment was conducted to check for oxygen contamination. The details of the multidisciplinary cruise onboard RV Sindhu Sadhana are given in Table S1. Viral lytic production was conducted from water that was collected from a depth of 146 m, with an oxygen concentration of 11.2 µM. Samples were collected separately for oxygen measurement at different intervals to check for oxygen contamination. The amount of oxygen contamination was 4.47 ± 2.18 µM from the beginning (t_0_) and end (t_72_) of the experiment. The plastic syringes might not be completely air tight allowing variations in oxygen levels. In the present study, the water column was classified into three distinct layers, the oxic layer (141.95 ± 28.97 µM), oxycline (69.78 ± 12.47 µM), and the hypoxic to suboxic bottom layers (12.47 ± 3.38 µM). Though there were variations in oxygen contamination, it was not sufficient to shift enough to increase the oxic status of the water samples outside of our predefined ranges. The prokaryotic abundance also remained stable over time suggesting oxygen contamination did not affect cell abundance. Thus, we assume that the oxygen contamination was negligible in the present study. The VP rates generally declined with a decrease in oxygen concentration and it ranged from 0.18 to 1.79 × 10^9^ VLPs L^−1^d^−1^ (Figure 5, Figure S3). VP rates were high in the surface waters (1.12 to 1.79 × 10^9^ VLPs L^−1^d^−1^), but it decreased significantly (by 67%) in the oxycline. Lytic viral production was undetectable in the hypoxic/suboxic waters. The high VP rates at the surface waters were comparable with the Mediterranean Sea and Baltic Sea [19].

Burst size (BS) estimates, defined as the number of viral particles released from a prokaryotic cell upon successful lysis are crucial for calculation of viral production. BS has been reported to range from 6 to 300 in marine systems and from 4 to 140 in freshwater systems [56]. The decrease in BS with decreasing DO concentrations explains the lower calculated viral lytic production rates in oxycline and hypoxic waters. BS was higher in the surface waters (21–31 viruses per prokaryote) as compared to lower estimates in the oxycline (17–21 viruses per prokaryote) and it was undetectable in the hypoxic/suboxic waters. Significant correlation between frequency of infected cells and burst size suggest that bacterioplankton host populations produced greater numbers of viruses under environmental conditions favoring fast growth and high productivity. Therefore, prokaryotic activity might be a driving force for viral lysis, which could be more beneficial for viruses to lyse a cell, eventually increasing the number of progeny and hence the probability of new infection [39,57].

The low VP rates could also be due to a reduced/lack of infection of viruses. The percentage of viral infection (FIC), which ranged from 8.2–10.3% in the surface waters, decreased by 50% in the oxycline and to undetectable limits in hypoxic/suboxic waters. Low viral infection rates and viral production in hypoxia coincided with the low availability of suitable host (low prokaryotic abundances). It has been observed that a minimum host cell concentration of 5 × 10^5^ cells mL^−1^ is a pre-requisite for successful viral replication [58]. Data from northern Adriatic seawater samples also indicated that no lytic infection occurred when the number of prokaryotic hosts fell below 2 × 10^5^ cells mL^−1^ [59]. The requirement of a minimum host density for efficient viral propagation has also been demonstrated from cyanophages in coastal seawaters [60]. Low virus-to-prokaryote ratio, together with low infection and low viral-induced mortality, could be responsible for the low VP rates in oxygen deficient waters.

**Figure 6:**
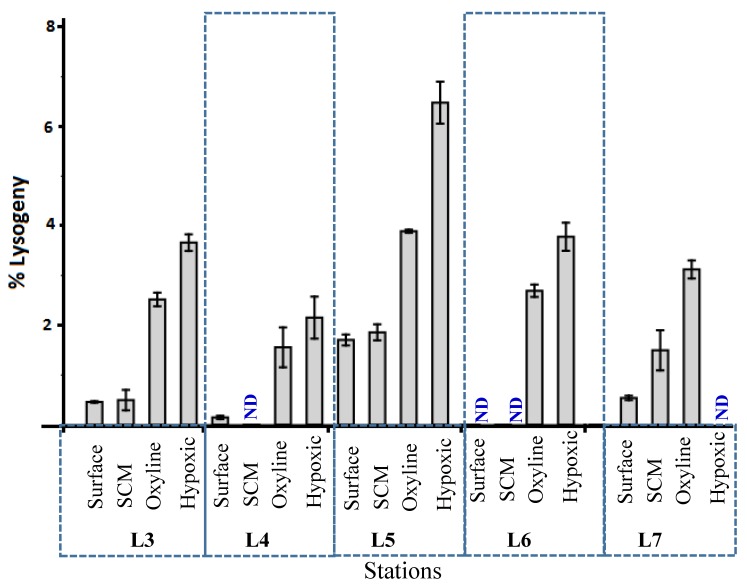
Percentage lysogeny at various oxygen concentrations, surface, SCM, oxycline, and hypoxic layers at stations L3, L4, L5, L6 and L7.

Overall, in this study, the percentage of lysogeny determined by using an inducing agent, mitomycin C, ranged from undetectable to 48% (Figure 6). Lysogeny was undetectable to 1.7% in oxic waters, whereas it was higher in oxycline (9–24%) and maximum in the hypoxic to suboxic waters (24–48%). We report the existence of contrasting pattern in viral reproductive strategy, which is significantly correlated to dissolved oxygen concentrations. Our finding agrees with the notion that lysogeny is a survival and maintenance strategy for viral populations that are threatened by harsh nutrient/host conditions and therefore, cannot sustain population numbers through lytic infection alone [61]. Lysogenic adaptation provides a refuge for viruses inside the prokaryotic cell. Lysogeny can favour transfer of viral genes between hosts by transduction [54]. Knowledge on biotic and abiotic factors in oxygen-limited environments that set the lysogeny-to-lytic switch will definitely shed new light on the role of temperate viruses in the microbial food web and energy transfer to higher trophic levels [62].

**Figure 7:**
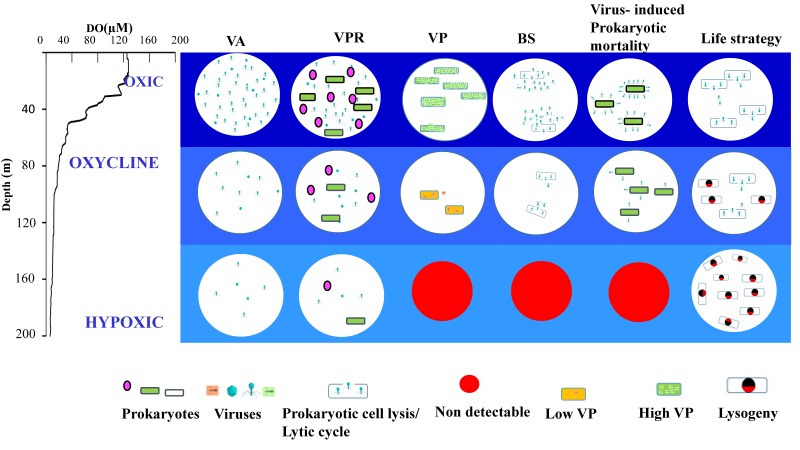
Conceptual diagram showing variations in viral parameters at different oxygen concentrations. The variations in viral parameters such as viral abundance (VA), virus to prokaryote ratio (VPR), viral production (VP), burst size, viral mediated prokaryotic mortality and reproductive life strategies at various vertical layers, oxic, oxycline, and hypoxic waters are represented.

To summarize, distinct viral life strategies were observed in the three layers of the vertical water column, namely, the oxic, oxycline and hypoxic waters, which is better represented in a flow diagram (Figure 7). Viral characteristics in the surface waters were typical of other oceanic waters with high viral abundances, virus-to-prokaryote ratios, and production rates. In the oxycline, both lytic and lysogenic reproduction strategies were found to co-exist. In the hypoxic layer, lytic activities were less evident, while lysogeny seemed to be a major strategy for viral survival. The frequency of lysogenised cells (FLC) increases from the coast to offshore and from surface to deeper waters [19,63,64]. Higher percentages of lysogeny has been reported from oligotrophic conditions owing to low host density and low nutrient conditions [63,65,66,67,68]. Lysogeny provides a temporary refuge for viruses when hosts are nutrient starved and scarce suggesting a “low host density-lysogenic dynamics” condition [65]. The lysogenic mode of reproductive cycle is favoured under poor prokaryote cell abundance, typically under 10^5^ cells mL^−1^ [66]. The average prokaryotic abundance in oxycline and hypoxic waters were 6 ± 3 × 10^4^ cells mL^−1^ and 2 ± 2 × 10^4^ cells mL^−1^ respectively. Hence, our study supports the hypothesis that lysogeny could be a survival strategy of viruses in deeper waters with low host cell abundance. A decrease in BS and FIC can be taken as evidence of the mechanism of a decrease in VA, which in our study explained decreased importance of viral lysis along the oxygen gradient. In addition to host factors, environmental variables also have significant influence in describing viral abundances across wide-ranging marine environments [69]. The changes in activity, diversity, and distribution of prokaryotes with the expansion of oxygen-depletion will likely determine the shifts in viral life strategies and their assemblages [17]. Hence, the abundance and production rates of viruses in oxygen limited waters should largely depend on microbial metabolism and assemblage composition.

## 4. Conclusions

Viral processes are understudied in oxygen deficient systems. To our knowledge, only a few studies have focused on the viral processes in OMZs [14,15,18,19]. In the present study, we show distinct patterns in viral abundance, activities, and contrasting life strategies along the vertical dissolved oxygen gradients in the Arabian Sea. Variations in virus abundance and infection rates are suggestive of their significant role in the microbial food web. Unlike high viral production in the surface waters, the low virus-to-prokaryote ratio in oxygen-deficient waters suggests decreased viral production and lytic infection, owing to reduced prokaryotic abundance. Interestingly, the above phenomenon was explained by a shift in the viral reproductive strategy from lytic to lysogenic in hypoxic waters. Depending on the predominance of lysogenic or lytic infection, the specific role of viruses from ecological and biogeochemical perspective may perhaps differ along oxygen gradients. Future research should focus to elucidate the ecological role of lytic and lysogenic life strategies in Arabian OMZs.

## Figures and Tables

**Table 1:** Table showing the positions of the stations sampled during this study. Three stations were sampled during 2015 and five stations were sampled during 2016. Station L4 was sampled during both years.

Sl. No	Latitude	Longitude	Location Name	Depth (m)
***RV Sindhu Sadhana* (2015)**				
1	15.3005 N	72.82274 E	L1	200 m
2	12.56856 N	73.75 E	L2	400 m
3	9.956254 N	75.54024 E	L4	200 m
***RV Sindhu Sankalp* (2016)**				
5	10.7028 N	75.5691 E	L3	200 m
6	9.956254 N	75.54024 E	L4	200 m
7	9.76388 N	75.61861 E	L5	200 m
8	8.13944 N	76.7325 E	L6	200 m
9	7.93388 N	77.9722 E	L7	200 m

**Table 2:** Table showing the Mean ± SD of various biotic and abiotic variables measured at surface, surface chlorophyll maxima (SCM), oxycline, and hypoxic waters in the present study. Abbreviations used: viral abundance (VA), prokaryotic abundance (PA), virus to prokaryote ratio (VPR), viral production (VP), Chlorophyll *a* (Chl *a*), dissolved oxygen (DO), nitrate (NO_3_), Nitrite (NO_2_), Phosphate (PO_4_), and silicate (SiO_4_).

Parameters	Surface	SCM	Oxyline	Hypoxic
Depth (m)	5.0 ± 2.4	29.0 ± 12.1	64.0 ± 10.2	200 ± 10.1
Temperature (°C)	30.1 ± 0.4	29.3 ± 1.21	20.8 ± 2.0	14.6 ± 2.0
Salinity	35.5 ± 2.4	36 ± 2.4	35.3 ± 2.49	35.2 ± 2.6
DO (µM)	146.3 ± 29.0	137.6 ± 28.2	69. 8 ± 12.5	12.5 ± 3.4
NO_2_ (µM)	0.09 ±0.10	0.24 ± 0.23	0.11 ± 0.10	0.08 ± 0.11
NO_3_ (µM)	1 ± 1.8	0.6 ± 0.8	6.2 ± 5.7	7.8 ± 3.7
PO_4_ (µM)	0.4 ± 0.5	0.4 ± 0.3	1.0 ± 0.5	1.7 ± 0.7
SiO_4_ (µM)	3.3 ± 4.5	3.2 ± 2.8	10.1 ± 6.3	17.8 ± 8.6
Chl *a* (mg/m^3^)	0.3 ± 0.3	0.6 ± 0.4	0.1 ± 0.03	0.03 ± 0.03
PA (10^5^ Cells/mL)	1.6 ± 1.1	1.6 ± 1.1	0.6 ± 0.3	0.2 ± 0.2
VA (10^6^ VLPs/mL)	6.1 ± 3.4	4.7 ± 2.9	1.2 ± 0.5	0.3 ± 0.3
VPR	4.4 ± 0.8	4.2 ± 2.4	2.3 ± 1.0	1.8 ± 0.8
VP (10^9^ VLPs/L/d)	1.1 ± 0.4	0.2 ± 0.1	ND	ND

**Table 3:** Results of the DistLM (non-parametric distance-based linear regression analysis) for understanding the effects of physico-chemical parameters on biological variables explaining VA.

Variable	SS	F^P^	Pc	Prop.
DO	6738.6	86.388	0.001 *	0.74867
NO_2_	549.65	1.8862	0.156	6.11E-02
NO_3_	5234.5	40.306	0.089	0.58157
PO_4_	4901.2	34.671	0.079	0.54453
SiO_4_	4381.9	27.512	0.002	0.48684
Temp	6421.8	72.214	0.001 *	0.71348
Salinity	617.62	2.1366	0.136	6.86E-02
PA	3237.7	16.293	0.001 *	0.35972
Chl *a*	4187.8	25.233	0.001 *	0.46527

Bold values indicate significant * *p*-values (*p* = 0.001). The abbreviations used are DO: Dissolved oxygen, NO_2_: Nitrite, NO_3_: Nitrate, NH_4_: Ammonia, PO_4_: Phosphate, SiO_4_: Silicate VA: Viral abundance, PA: Prokaryotic abundance, Chl *a*: Chlorophyll *a*. F^P^: Pseudo F-values, Pc: *p*-values, SS (Trace): portion of sum of squares relative to the analyzed predictor variable and prop: proportion of variation explained by the explanatory variables.

## Supplementary Materials

The following are available online at http://www.mdpi.com/1999-4915/10/11/588/s1.
