# Supplementary material for: High Incidence of Lysogeny in the Oxygen Minimum Zones of the Arabian Sea (Southwest Coast of India)
Source: Viruses. 2018 Oct 27;10(11):588. doi: 10.3390/v10110588 (PMC6267222; doi:10.3390/v10110588)
Supplement: Supplementary file 1 [file viruses-10-00588-s001.pdf]

(a)

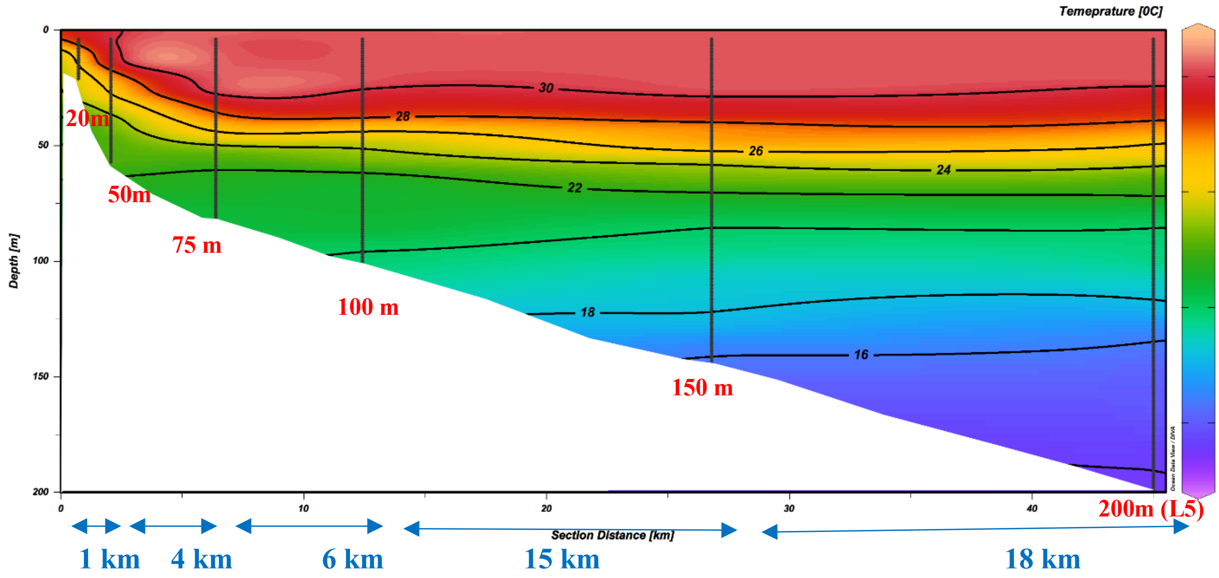

(b)

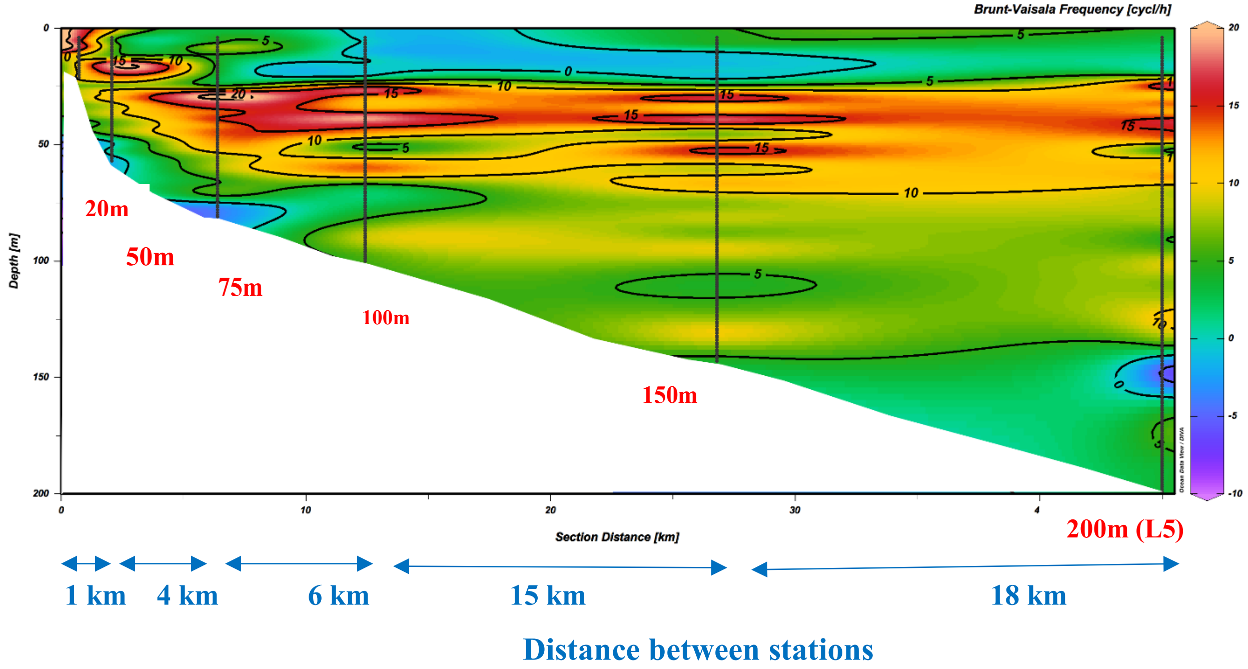

Supplementary Figure 1 (a) Represent the temperature-depth profile along a transect containing station L5. Samples were taken at different depths, 20 m, 50 m, 75 m, 100 m, 150 m and 200 m (L5). X- axis represents the station locations and Y-axis represent the depth of the stations in the transect stationing L5, and Z-axis represent temperature profile in color contours. The vertical section of temperature indicates upwelling signature at L5. (b) Brunt-Vaisala frequency plot for water column stability demonstrating upwelling. Highly stable water column (>10 cycles/hr) was noticed in the thermocline region with an upslope towards the surface indicating strong upwelling in this region. The temperature also showed upsloping of isotherms towards the coastal region

under the influence of strong Ekman pumping. The plots were generated using the Ocean Data View software package (version 4.7.10) which is available freely. Red marking indicates the depth points and blue marking indicates the distance between stations.

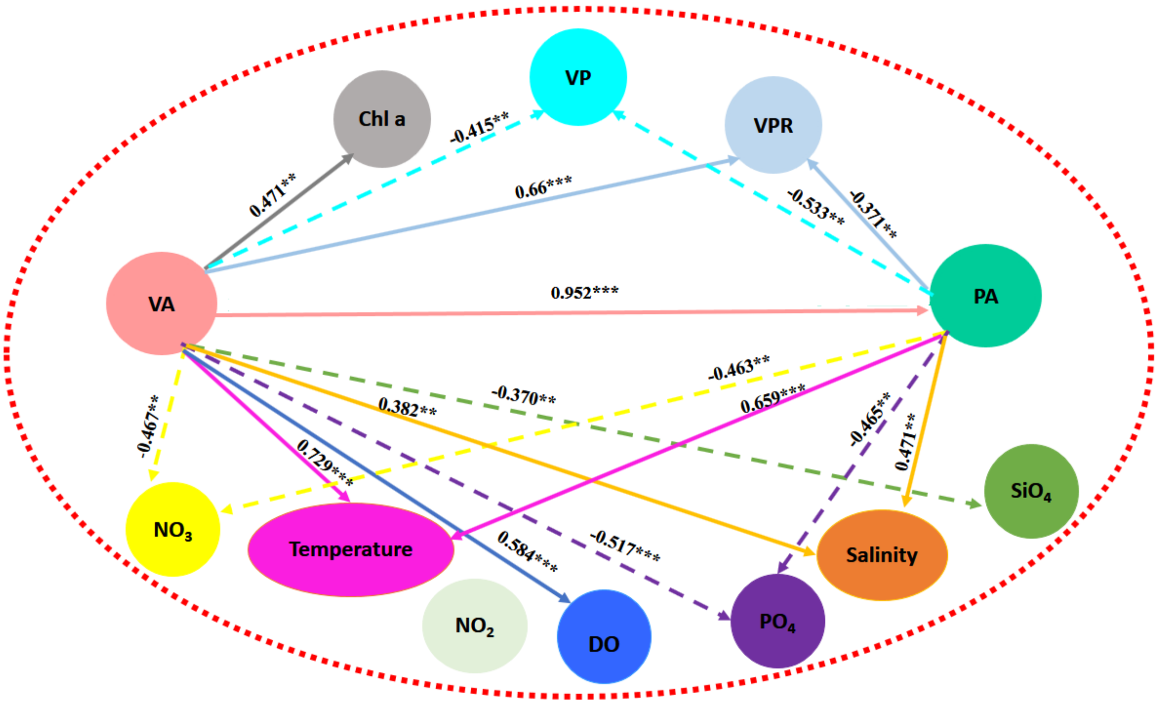

Supplementary Figure 2 Schematic diagram showing the two-tailed correlation of the abundance of viruses and prokaryotes with physicochemical and biological parameters. The parameters with significant positive correlation are connected through lines and negative correlation with dotted lines. The coefficient of correlation (r value) are given along the lines and *indicates significant r- values at *p < 0.1, **p < 0.05 and ***p < 0.01.

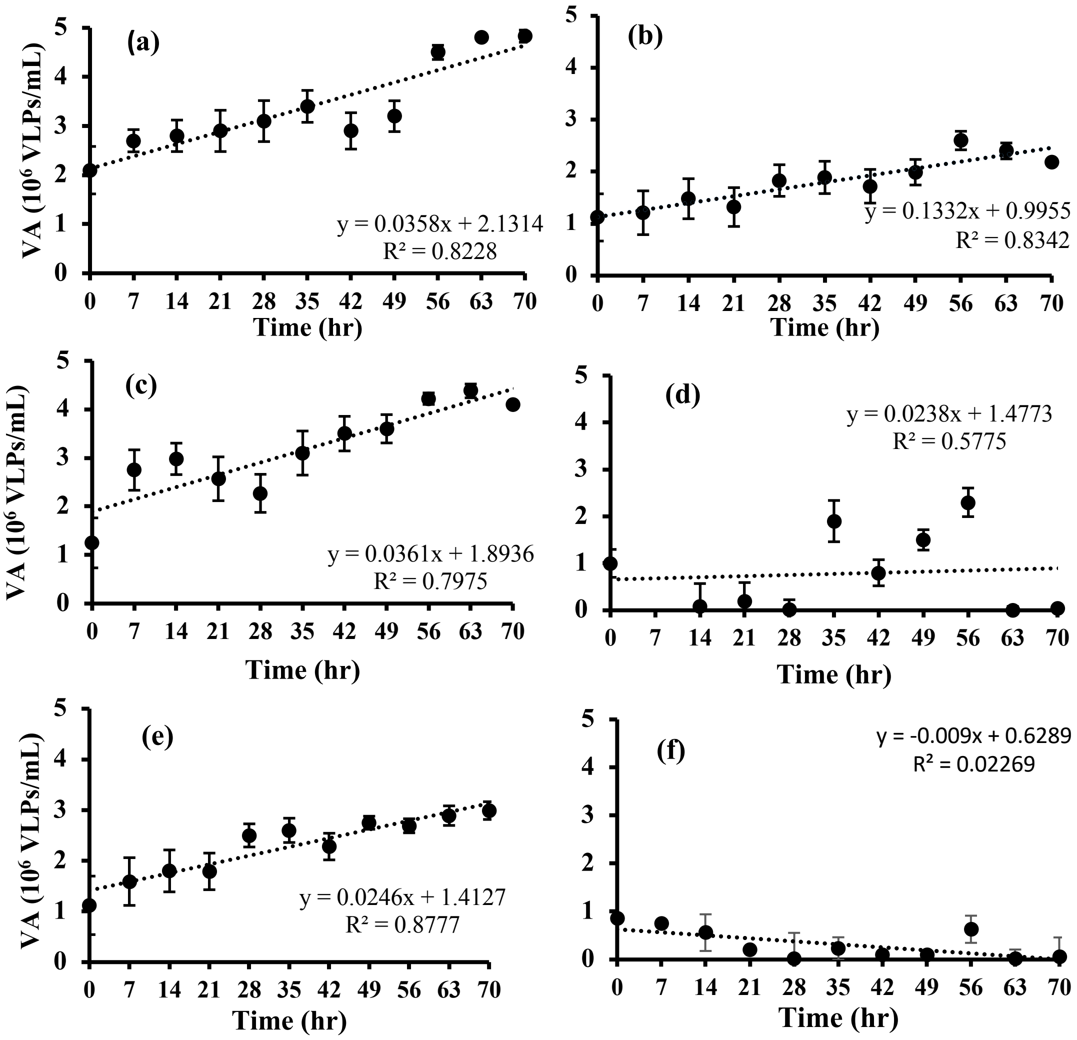

Supplementary Figure 3 represents the viral lytic production at (a) L3, (b) L4, (c) L5, (d) L6 (e) L7 and (f) ND plot. Dotted line represents the regression line with time in hours. Error bars are standard deviation of the mean of 3 replicates

Cruise No: SSD 049	
Latitude	16.024519° N
Longitude	81.824532° E
Total Depth (m)	706.2 m
Sampling depth	146 m
Temperature (°C)	12.15 ±1.12
Salinity	35.18 ± 2.18
**DO (µM) (t0)**	**11.20 ± 2.17**
**DO (µM) (t48)**	**12.15 ± 2.12**
**DO (µM) (t72)**	**15.67 ± 1.12**
NO_2 (µM)	0.12 ± 0.6
NO_3 (µM)	5.22 ± 0.89
PO_4 (µM)	1.18 ± 0.59
SiO_4 (µM)	3.4 ± 2.89
Chl *a* (mg/m^3)	0.02 ± 0.04
PA (10^5 Cells/ml)	0.2 ± 0.04
VA (10^6 VLPs/ml)	0.3 ± 0.02

Supplementary Table 1. Details of viral production experiment conducted in the hypoxic waters (depth, 146 m, dissolved oxygen 11.47 µM) during a multidisciplinary oceanographic cruise onboard, RV Sindhu Sadhana. Mean ± SD of various biotic and abiotic variables at this hypoxic layer is indicated in the table. Abbreviations used: viral abundance (VA), prokaryotic abundance (PA), Chlorophyll *a* (Chl *a*), dissolved oxygen (DO), nitrate (NO_3), Nitrite (NO_2), Phosphate (PO_4), and silicate (SiO_4). The dissolved oxygen (DO) concentrations at T0, T48 and T72 is represented.
